# Photobiomodulation in dental implant stability and post-surgical healing: A clinical study

**DOI:** 10.6026/973206300212369

**Published:** 2025-08-31

**Authors:** Gousia Shafat Khan, Syed Aaliyah, Uday Sagar Sandepogu, Jignesh Tate, Rahul Tiwari, Prashant M.C., Heena Dixit Tiwari

**Affiliations:** 1 Department of Prosthodontics, Government Dental College and Hospital Srinagar, Srinagar, Jammu And Kashmir, India; 2 Department of Prosthodontics, Crown & Bridge, Government Dental College and Hospital Srinagar, Jammu And Kashmir, India; 3 Department of Oral and Maxillofacial Surgery, Consultant Maxillofacial Surgeon and Implantologist, St Theresa Hospital, Hyderabad, India; 4 Department of Periodontics, Bharati Vidyapeeth Dental College and Hospital, Navi Mumbai, Maharashtra, India; 5 Department of Oral and Maxillofacial Surgery, RKDF Dental College and Research Centre, Sarvepalli Radhakrishnan University, Bhopal, Madhya Pradesh, India; 6 Department of Oral and Maxillofacial Surgery, RKDF Dental College and Research Centre Bhopal, Madhya Pradesh, India; 7 Commisionerate of Health and Family Welfare, Government of Telangana, Hyderabad, India

**Keywords:** Photobiomodulation (PBM), dental implants, implant stability, soft tissue healing, low-level laser therapy

## Abstract

Photobiomodulation also known as low-level laser therapy has emerged as a non-invasive approach to enhance post-surgical outcomes by stimulating cellular activity and promoting tissue regeneration. This randomized clinical research included 40 patients requiring single-tooth implants in the posterior mandible. PBM significantly improved ISQ values at all post-operative time points (p < 0.01). Soft tissue healing scores were also superior in the PBM set (p < 0.05). No adverse events occurred. PBM significantly enhances early implant stability and accelerates soft tissue healing, supporting its use as an adjunct in dental implant therapy.

## Background:

Dental implantology has become a cornerstone of modern restorative dentistry, offering reliable and long-term solutions for tooth replacement. While the success rate of dental implants has improved significantly over the years, achieving optimal osseointegration and minimizing post-surgical complications remain critical goals in implant therapy. In this context, PBM therapy-also referred to as "low-level laser therapy (LLLT)"-has garnered increasing attention as a non-invasive adjunct capable of enhancing tissue healing and implant stability [1]. PBM employs low-power light energy, typically in the red or near-infrared spectrum, to stimulate cellular activity and accelerate biological responses. The mechanism involves the absorption of photons by mitochondrial chromophores, leading to increased "adenosine triphosphate (ATP)" production, modulation of "reactive oxygen species (ROS)" and the activation of transcription factors that promote cellular proliferation and repair [2, 3]. This biochemical cascade has been shown to positively influence bone remodeling, angiogenesis and inflammation control-key determinants of implant success [4, 5]. The immediate post-operative phase is particularly crucial, as it determines the quality of early healing and the potential for long-term osseointegration. Several clinical trials and animal studies have suggested that PBM may enhance implant stability by improving bone-implant contact, reducing soft tissue edema and accelerating collagen matrix formation [6, 7-8]. Moreover, PBM has demonstrated analgesic properties and may reduce reliance on systemic pain medications, thereby improving patient compliance and comfort [9]. Despite growing evidence, the integration of PBM into routine implant protocols is still not standardized, partly due to variability in laser parameters, treatment timing and lack of uniform guidelines. Hence, further clinical investigation is warranted to validate its efficacy and establish optimized protocols for different patient demographics and surgical conditions [10]. Therefore, it is of interest to assess the impact of PBM on implant stability and soft tissue healing in a clinical setting, thereby contributing meaningful data to bridge the current evidence gap.

## Materials and Methods:

This prospective, randomized clinical research was conducted to assess the effects of PBM on implant stability and soft tissue healing following dental implant placement. Ethical clearance and informed consent was secured from all participants prior to enrolment.

## Research population:

A total of 40 systemically healthy adult patients (aged 25-60 years) requiring single-tooth dental implants in the posterior mandible were selected. Patients were randomly divided into two sets: (1) Set A (PBM Set, n=20): Received PBM therapy post-surgery, (2) Set B (Control set, n=20): Received standard post-operative care without PBM. Inclusion criteria included good oral hygiene, adequate bone volume (≥6 mm width, ≥10 mm height), and non-smokers. Exclusion criteria were systemic diseases affecting bone metabolism (e.g., diabetes, osteoporosis), pregnancy, use of bisphosphonates or corticosteroids, and poor oral hygiene.

## Surgical procedure:

All implants used were titanium, self-tapping, and of the same design (4.2 mm x 10 mm). Implant placement was performed under local anesthesia using a standard two-stage surgical protocol. All procedures were carried out by the same experienced oral surgeon to minimize technique variability.

## PBM protocol:

In set A, PBM was administered immediately after surgery and on post-operative days 3, 7, and 14 using a diode laser device (wavelength: 810 nm; power: 100 mW; energy density: 6 J/cm² per site; application time: 60 seconds per point). Laser was applied at four sites per implant (buccal, lingual, mesial and distal) in a non-contact mode.

## Outcome measures:

Implant stability was assessed using resonance frequency analysis (RFA) to calculate the Implant Stability Quotient (ISQ) values at baseline (implant placement), 2 weeks, 4 weeks, and 8 weeks. Soft tissue healing was evaluated using the Landry Wound Healing Index on days 7 and 14 post-operatively. Data were analyzed using SPSS version 25.0. A p-value of <0.05 was considered statistically significant.

## Results:

A total of 40 patients completed the research, with 20 in each set (PBM and control). No patients were lost to follow-up. Healing was uneventful in all participants, and no implant failures were recorded during the 8-week observation period. At baseline (immediate post-placement), mean ISQ scores were comparable between both sets (PBM: 67.5 ± 2.4 vs. Control: 66.9 ± 2.6; p = 0.38). By week 2, set A (PBM) showed a statistically significant increase in ISQ values compared to set B (71.4 ± 2.5 vs. 68.1 ± 2.9; p = 0.002). This trend persisted at weeks 4 and 8, where ISQ values in the PBM set were significantly higher, suggesting enhanced osseointegration (Table 1 - see PDF). The PBM set showed better healing outcomes at both day 7 and day 14. On day 7, 85% of patients in set A demonstrated "excellent" healing compared to 55% in set B (p = 0.03). By day 14, all patients in set A achieved "excellent" healing, while only 70% in set B did (p = 0.01) (Table 2 - see PDF).

## Discussion:

The present research assessed the impact of PBM on the stability of dental implants and the quality of post-surgical soft tissue healing. The findings clearly indicate that PBM significantly enhances both implant stability and healing outcomes compared to conventional post-operative protocols. In terms of implant stability, a progressive increase in ISQ values was observed in the PBM set over the 8-week period, with statistically significant differences beginning from the second week. This trend supports earlier findings that PBM facilitates early bone remodeling by stimulating osteoblastic proliferation, increasing vascularity, and enhancing mitochondrial activity in peri-implant bone tissue [11]. The red to near-infrared light spectrum used in PBM is absorbed by cytochrome c oxidase, which catalyzes ATP production, thereby promoting cellular energy availability for tissue regeneration [12]. The observed acceleration in soft tissue healing further substantiates the biological rationale behind PBM use. Improved healing scores in the PBM set on days 7 and 14 suggest that the therapy enhances collagen synthesis and reduces inflammatory markers, creating a more favorable environment for tissue repair [13]. This is consistent with the known anti-inflammatory effects of LLLT, which has been shown to modulate the expression of prostaglandins, interleukins, and other cytokines in wound healing models. Another noteworthy observation was the absence of implant failures or complications in both sets, reflecting the controlled surgical conditions and standardized implant systems used. However, the difference in soft tissue healing quality implies that PBM not only accelerates the healing timeline but may also improve tissue resilience in the critical early post-operative phase. Despite promising results, certain limitations should be acknowledged. The research was conducted on single-implant cases in healthy individuals, which may not reflect outcomes in compromised patients or multiple implant situations. Furthermore, while the PBM parameters used were effective, different dosimetries may yield varying outcomes, indicating a need for protocol standardization in future research [14]. Clinical adoption of PBM remains variable due to lack of regulatory guidance and clinician awareness. Nonetheless, the current findings provide further justification for its integration into dental implant protocols, particularly in cases requiring enhanced healing or faster loading timelines [15, 16, 17, 18, 19-20].

## Conclusion:

The use of LLLT led to faster osseointegration as evidenced by higher ISQ values and enhanced wound healing scores. These results support the inclusion of PBM as a non-invasive adjunct in implant dentistry, particularly for optimizing early healing outcomes. PBM shows great potential to become a valuable tool in improving the predictability and success of dental implant procedures.

## References

[1] Camolesi GCV (2023). Clin Oral Implants Res..

[2] Bozkaya S (2021). Clin Implant Dent Relat Res..

[3] Saini RS (2024). Photodiagnosis Photodyn Ther..

[4] Collado-Murcia Y (2024). J Clin Med..

[5] Vande A (2022). Dent Med Probl..

[6] Sourvanos D (2023). Photobiomodul Photomed Laser Surg..

[7] Foletti JM (2023). J Dent Res Dent Clin Dent Prospects..

[8] Poli PP (2022). Materials (Basel)..

[9] Gulati P (2020). Clin Implant Dent Relat Res..

[10] Okuhama Y (2022). BMC Oral Health..

[11] Supachaiyakit P (2022). Clin Implant Dent Relat Res..

[12] Palled V (2021). J Oral Implantol..

[13] Eid FY (2022). BMC Oral Health..

[14] Hakimiha N (2021). Quintessence Int..

[15] Shenoy A (2024). Cureus..

[16] Mehjuba (2025). J Pharm Bioallied Sci..

[17] Perti S (2024). J Pharm Bioallied Sci..

[18] Mahmoud ES (2025). Head Face Med..

[19] Chen Y (2019). BMC Oral Health..

[20] Mandal NB (2022). J Pharm Bioallied Sci..

